# New York County

**Published:** 1899-05

**Authors:** Robert W. Ellis

**Affiliations:** Secretary


### VETERINARY MEDICAL ASSOCIATION OF NEW YORK COUNTY.

The regular monthly meeting was called to order at the New York Academy of Medicine, 17 West Forty-third Street, at the usual hour, April 6th, by President Robertson. The following members responded to roll-call: Drs. Bretherton, Bell, Clayton, Dickson, Ellis, Hanson, Keller, MacKellar, O'Shea, and Robertson. Visitors: Drs. Howe, Ohio, and Nicolas, New York. The minutes of the previous meeting were next read and approved.

Dr. Clayton, chairman of the Board of Censors, reported that the committee favorably recommended for membership in the Association Dr. C. H. DuBois, graduate of the American Veterinary College, class of 1896, whose application had been filed at the last meeting. Moved and seconded, that the report of the committee be accepted, and that Dr. DuBois be declared a member of the Association; carried. Dr. DuBois was then introduced to the members by Dr. Clayton.

Dr. R. S. MacKellar then read a paper entitled "Paraldehyd in Veterinary Practice." Dr. MacKellar's paper, brief and to the point, was listened to with keen interest by his fellow-members, who gave expression to their interest in the discussion which followed. Dr. Bell thought that the use of paraldehyd in veterinary practice a revelation to most veterinarians; he had never heard of its use in veterinary practice for this purpose, therefore thought this paper of great importance, as we may profit by the experience of the essayist. He considers it a great advantage, as it permits us to do many operations standing in cases where we could not, possibly, have obtained the owner's permission to cast the animal. Dr. Hanson said that in human practice paraldehyd is considered inferior to chloral, and asked if the essayist found it superior. Dr. MacKellar said he regarded it as superior for the purpose used.

Dr. Clayton asked if the effects upon the motor nerves were less than with chloral, stating that it had been recommended to him for castrating standing, and asked how long its effects persist.

Dr. MacKellar stated that its effects on the motor nerves are less than with chloral, and persist for about an hour.

Dr. Clayton asked if there were any bad after-effects the next day.

Dr. MacKellar said that there were none.

Dr. Nicolas asked if hemorrhage was less profuse than when chloral is used.

Dr. MacKellar said he had not noted any difference, but was not prepared to state positively.

Dr. Clayton asked if it at all retarded the healing process.

Dr. MacKellar said no. Dr. Ellis asked if the essayist had experimented with it in colic. Dr. MacKellar said no; but in reading up on it in Finlay Dunn, he learned that it has no effect on internal pain. Dr. Bell said to depart from paraldehyd for a moment, he would like to know the experience of some of the members present on the use of acetanilid. Dr. MacKellar said he used it in a case of pneumonia with temperature of 107° F.; horse so weak that he fell when man laid his hand heavily upon him (man being intoxicated), and got marked improvement from its use. Dr. Bell said that he was very enthusiastic over the results obtained from the employment of this drug, and clinical experience, with him at least, has disproved the charge against it as a heart-depressor. He considers eight hours the proper period of time that should elapse between doses, allowing the temperature to slightly elevate after the effects of the last dose, and then repeat, and considers two drachms to be the proper dose in conjunction with digitalis. Dr. Clayton asked if the same results could be obtained from a smaller dose than two drachms. Dr. Bell said, no; you must give two drachms to get the results, and for rheumatoid fevers it is as near magical as anything in the drug line. Dr. Howe said that he did not come up here to say anything, but was interested in this discussion; a favorite prescription with him for influenza is a combination of acetanilid, quinine and digitalis.

Moved by Dr. Hanson and seconded by Dr. O'Shea, that a vote of thanks be extended to Dr. MacKellar for his interesting and valuable paper; carried.

The committee to investigate the legality of the appointment of laymen as meat-inspectors (Dr. Hanson, chairman) read a letter from the President of the Health Department, in which he states that while he does not doubt the correctness of the preamble and resolutions contained in Dr. Hanson's communication of February 7th, the department under the present law is compelled to take from the list certified to it by the Civil Service Commission, and has no voice or choice in the matter. Moved by Dr. Clayton, that the chairman of this committee send a registered letter to the Secretary of the Civil Service Commission, calling his attention to the former letter; seconded; carried.

Dr. Robertson reported a case of the sudden death of a young, green horse—falling dead after ascending a hill—in which post-mortem revealed hemorrhage as the cause of death, due to rupture of a large vessel; but as the post-mortem had to be held by the light of a lantern, they did not learn what vessel it was. Dr. Clayton reported several cases of sudden death that had come under his notice: One while scoring heats on a race-track, another in which death occurred while performing neurotomy, the animal being cast; post-mortem revealed heart-rupture; and a third one suffering with purpura hemorrhagica.

Dr. Bell moved that as an adjournment to Dr. Clayton's paper on median neurectomy, read at the last meeting, he perform the operation upon a subject which he would furnish. After some discussion it was regularly moved and seconded that the Secretary notify the members of the Association that a surgical clinic would be held at the American Veterinary College on Tuesday, April 11th, at 4 P.M.; carried.

Dr. Bell, chairman of the Ways and Means Committee, reported that there would be two papers at the next meeting and a surgical clinic in the interim.

Moved and seconded that the meeting adjourn; carried.

ROBERT W. ELLIS, D.V.S.,
Secretary.

